# Teamwork in primary palliative care: general practitioners’ and specialised oncology nurses’ complementary competencies

**DOI:** 10.1186/s12913-018-2955-7

**Published:** 2018-03-07

**Authors:** May-Lill Johansen, Bente Ervik

**Affiliations:** 1 Department of Community Medicine, UiT The Arctic University of Norway, N-9037 Tromsø, Norway; 2 Department of Health and Care Sciences, UiT The Arctic University of Norway, Tromsø, Norway; 3 Department of Oncology, University Hospital of Northern Norway, Tromsø, Norway

**Keywords:** Palliative care, General practice, Interprofessional, Teamwork

## Abstract

**Background:**

Generalists such as general practitioners and district nurses have been the main actors in community palliative care in Norway. Specialised oncology nurses with postgraduate palliative training are increasingly becoming involved. There is little research on their contribution. This study explores how general practitioners (GPs) and oncology nurses (ONs) experience their collaboration in primary palliative care.

**Methods:**

A qualitative focus group and interview study in rural Northern Norway, involving 52 health professionals. Five uni-professional focus group discussions were followed by five interprofessional discussions and six individual interviews. Transcripts were analysed thematically.

**Results:**

The ideal cooperation between GPs and ONs was as a “meeting of experts” with complementary competencies. GPs drew on their generalist backgrounds, including their often long-term relationship with and knowledge of the patient. The ONs contributed longitudinal clinical observations and used their specialised knowledge to make treatment suggestions. While ONs were often experienced and many had developed a form of pattern recognition, they needed GPs’ competencies for complex clinical judgements. However, ONs sometimes lacked timely advice from GPs, and could feel left alone with sick patients. To avoid this, some ONs bypassed GPs and contacted palliative specialists directly.

While traditional professional hierarchies were not a barrier, we found that organization, funding and remuneration were significant barriers to cooperation. GPs often did not have time to meet with ONs to discuss shared patients. We also found that ONs and GPs had different strategies for learning. While ONs belonged to a networking nursing collective aiming for continuous quality improvement, GPs learned mostly from their individual experience of caring for patients.

**Conclusions:**

The complementary competences and autonomous roles of a specialised nurse and a general practitioner represented a good match for primary palliative care. When planning high-quality teamwork in primary care, organizational barriers to cooperation and different cultures for learning need consideration.

**Electronic supplementary material:**

The online version of this article (10.1186/s12913-018-2955-7) contains supplementary material, which is available to authorized users.

**How this fits in:** Little is known about how general practitioners and specialised nurses cooperate in primary palliative care. We found that ideally, in clinical care for particular patients, their competencies are complementary and should be joined. However, there were important organizational and cultural barriers to their cooperation, which need to be addressed.

## Background

Most palliative patients want to spend their end-of-life at home or close to home [1, 2], and many family members prefer to care for them at home [3]. Particularly in rural areas, generalists such as general practitioners (GPs) and district nurses (DNs) provide palliative home care. Most GPs see this as challenging, important and rewarding work [4–8]. Studies on how well GPs deliver palliative care have found a mixed picture [9]. Palliative care offered in teams of GPs and nurses had better clinical outcomes than that provided by GPs alone. GPs wanted more training in relieving pain and other symptoms [10, 11] and some nurses wished for improvement of GPs’ skills [12].

Publicly employed district nurses and small practices of usually self-employed GPs make up the backbone of primary care in Norway. The region of Northern Norway mainly consists of rural areas and a relatively small population of 500.000 people. Primary care is in the hands of 87 different local health authorities. Specialist care is mainly located in 11 hospitals, mostly small ones. About half of the local authorities have employed nurses with a postgraduate diploma in oncology nursing, including palliative care, hereafter called oncology nurses (ONs). They have become key workers in primary palliative care and in the supervision of district nurses [13]. Being specialised, their competence may be regarded as superior to that of GPs in some fields. Despite palliative home care requiring interprofessional collaboration, there is sparse research on such teamwork in the community [14].

GPs are often portrayed as if they were working solo with their palliative patients [15]; however, GPs often prefer to work in local palliative teams with nurses and other trusted care providers, if available [11, 16]. Hierarchical doctor-nurse relationships might persist in such teams [17], but not always [18]. There are few studies of how specialised nurses collaborate with, supervise and teach general practitioners, and vice versa [19–21]. Part of a larger study about rural palliative care, this paper aims to explore how rural GPs and ONs experience their roles and their collaboration in palliative care, including perceived barriers to their cooperation.

## Methods

To explore how rural GPs and ONs experience their collaboration in palliative care, we used focus group discussions (FGDs), held in 2015–2016 with health professionals involved in palliative care in rural communities of Northern Norway. The first five focus groups were held with nurses and physicians separately, in order to discuss their experiences of interdisciplinary working without the other group present. We had two groups of GPs and three groups of nurses, comprising both district nurses and oncology nurses. Each focus group had 3–8 participants. The first group of GPs was recruited by e-mail, and the FGD was held at a hotel. The second group of GPs was recruited by snowballing from the first, and held at the local hospital. Nurses were recruited by e-mailing participants on a palliative course and at palliative care network meetings, and the FGDs were held on the premises of these events. Geographically, the GPs and nurses came from 19 different local authority areas with a median population of 3028 inhabitants.

To obtain more detailed and contextual knowledge on local collaboration, six rural local authority areas were purposely selected for their different sizes, geography and organization of palliative care. BE asked the ON in each area to organize a group of professionals that would normally work together in palliative care. The participants were ONs, GPs, DNs and allied health professionals. In the first local area, six different professionals were interviewed individually at their health centre. Here, we wanted to gain a detailed picture of each professional in the palliative group, their contribution and their views on the joint collaboration. We then conducted FGDs in five other local areas, each with 3–7 professionals, aiming for discussion and interaction between the participants [22, 23] (Tables 1 and 2).

**Table 1:** Participants in the study

Profession	N	Age group	Gender
Oncology nurses	15	35–60	All women
District nurses	15	26–61	All women
General practitioners	17	27–68	6 men, 11 women
Allied health (physiotherapy, occupational therapy)	5	27–55	1 man, 4 women
Total	52

**Table 2:** Local authority areas represented

Profession	N of areas	Population size of areas
Nurse FGDs	14	1200–9600
GP FGDs	5	1200–50,000
Team FGDs + interviews	6	2000–8000
Total	25	

The interview guide consisted of a brief topic guide [24] (Additional file 1). We encouraged participants to share and discuss authentic personal experiences: critical incidents that could illuminate the research question. Such narratives create more rich and robust data than non-committal, general opinions [25]. The FGDs, lasting around 90 min, were mediated by BE and MLJ. BE is an ON by background while MLJ is an academic GP. Both have experience with qualitative methods from their PhDs and postdoc research. The individual interviews were performed by medical student BB, as part of her master’s thesis, supervised by MLJ. These interviews lasted around one hour. The ten FGDs and six interviews were digitally recorded and transcribed verbatim.

The authors undertook an inductive thematic analysis within a realist paradigm, and were interested in both semantic and latent content [26]. We coded the transcripts independently, looking for recurrent topics, patterns and “incidents that can illuminate the research questions” [25]. Potential overarching themes and subthemes were discussed between the researchers, revised and refined in an iterative process. All coded text on the final themes and subthemes was then extracted, joined and condensed. We aimed at illustrating subthemes with a rich selection of quotes, which also showed some of the interaction in the focus groups [23]. This paper reports on one of the four overarching themes: Teamwork as a joining of complementary competences. The other themes will be presented in subsequent papers.

To assure trustworthiness of our results, we used several strategies. For dependability, we kept an audit trail of the decisions during the analytic process. For transferability, we described characteristics of the research region, the involved local areas and our participants. For credibility and confirmability, we discussed our emergent findings with resource persons in the field, such as GPs, ONs and fellow researchers.

## Results

Several professions were involved in delivering primary palliative care. However, in this paper we will analyse in depth the cooperation between general practitioners (GPs) and oncology nurses (ONs). Drawing on participants’ stories, we will highlight characteristics of a well-working GP-ON relationship, unpick the missing elements in deficient collaborations, and identify barriers to effective cooperation.

## Complementary competencies

In local authorities with a long history of providing palliative home care, there had been a shift in roles for both GPs and ONs. While GPs did most of the work themselves 20 years ago, with the extended use of ONs, GPs now had a more advisory role. Moreover, with the development in recent years of palliative knowledge and experience amongst district nurses, ONs increasingly became advisors as well. Hence, both GPs and ONs could have expert roles within their local health care services.

There were some examples of close clinical cooperation around shared patients amongst our participants. These dyads often had a long-term professional relationship. Often palliative patients with worsening symptoms first talked to the ON, who then contacted the GP to discuss the case**.** The following example is seen from a GP’s perspective.

GPI1: *“And this cooperation, that she comes in to me and says that now this patient (…) is getting more pain (...) she has so much experience, then she says, “What if we increase for example… a fentanyl patch. Or we could add Haldol, or, couldn’t we? It often works well, I’d recommend it.” Because I know the patient, I can say, “That’s a good idea” and send a prescription to the pharmacy. Alternatively, I can say, “No, I, okay, but I have to look at her (...)” because it could be other causes, maybe radiation is indicated. Then...I put the patient somewhere into my schedule.”*

Hence, the ON contributed her competencies, which included the ability to accurately observe and describe symptoms and signs. Comparing this case to similar previous ones and drawing on her own experience and up-to-date knowledge of palliative care, she was able to make a treatment suggestion. However, she needed to discuss this. The GP drew on her previous relation to and knowledge of the patient, including knowledge about the resources of the patient and family.

GPI1: *“The most important part (of a GP’s skills) is knowledge about the patient, and knowledge about the whole picture of the patient’s illness. Not only the cancer, but also perhaps a previous heart attack, (and) a diagnosis of asthma. Well, there are so many things, many other things that the specialist maybe doesn’t know about, which I have information on.”*

Hence, the GPs drew on their generalist backgrounds, and the ON provided specialised palliative knowledge. While some of their competencies overlapped, GPs needed ONs to report from their longitudinal observations of the patient and make treatment suggestions, while the ON needed the GP to draw a diagnostic conclusion and write prescriptions. Cooperating closely sometimes meant seeing patients together, and in these situations, ONs and GPs could make use of their *complementary competencies*.

GPI1: *“Sometimes the ON joins me in the consultations with these patients, so that she gets all the information and we’re coordinated and agree, and we’ve discussed at the same time. I don’t always have all the answers. I often say to the ON during the consultation: What’s your opinion? (…) Then I kind of let her take control. (…) And I think that’s very important. To show it, I mean, to show it to the patient – that I don’t know everything”*.

## Deficient cooperation

Some ONs recalled situations where they did not achieve the necessary clinical discussions with the local GPs. It could be difficult to get hold of the GPs, to get timely call-backs or to get a medical opinion. ONs could feel left alone with a seriously ill patient, waiting for a solution.

ONT3: *“When we go out (on home visits), we’re quite alone. Well, not quite, I do have very good communication with you (doctors), but sometimes maybe – when I try to contact you, it’s because I need help there and then, not that I should have to wait for three or four hours perhaps. It can be very inconvenient because when I do a home visit and they’re very ill, well, it’s never happened that I didn’t get help, that’s true. But sometimes I do need some kind of answer to get on with things.”*

After the introduction of specialist palliative teams in local hospitals a decade ago, ONs could call the doctor there to get a second opinion if they were unsure about the quality or safety of the advice from the local GP.

ONT5: *“I ask them (local GPs) to consider this and that, and then they usually say that’s okay. No, it’s not always like that, but I have experienced it. I don’t think it’s quite safe. So then, I call the doctor on the specialist palliative team at the local hospital (…): Hi, I just need to discuss something a bit with you, because our doctors says yes to everything I ask for, which I don’t quite like. I need a discussion about it, you see. (…) Because I could be wrong.”*

Some ONs had even started to bypass the GPs. They called the specialists directly for advice, as they were regarded as more accessible and competent, and often knew the patient as well. A few GPs did not mind being bypassed, either because they were too busy anyway, or because they regarded palliative care as a small part of their portfolio. However, others felt that this meant that they lost (track of) their patients, which they saw as unsatisfactory and potentially unsafe.

Many ONs seemed to feel that the GPs were leaning on them and their palliative competencies. Being gently leant on could work well and give ONs recognition, as illustrated in this interprofessional FGD.

ONT4: *“Sometimes we feel that we’re the ones who say maybe it’s a good idea to do things like this or like that.”*

GPT4: *“Yes, that’s true. We’re happy with that. Yes.”*

ONT4: *“And that you feel reassured about… that we can dare to suggest things.”*

GPT4: *“You’re the ones working around the patient all the time, getting experience, and we might have nine or ten other things we have to relate to. So it’s very good that you do this training and can give us a bit of advice about what’s a good idea in this case and … yes.”*

However, if the GP leant too hard on the ON, the relationship got out of balance and the ON started to miss the support from the GP. The following excerpt is from an interdisciplinary discussion around the importance of doing home visits. The GP highlights the human connection to the patient, while the ON wants the doctor to consider the medical aspects as well.

GP1T3: *“It’s very important that we’re just there and chat a bit with them. Then they liven up, maybe they get some zest for life.”*

ON1T3: *“Yes and that part is very important too. But the medical part is also important, because they want the reassurance, and to know that the doctor who comes knows which medicines they’re taking and which dose and all that and they should….”*

GP1T3: *“Yes, yes. But… Yes, we do that, and… But you’ve often done it already when you’ve been there before us.”*

ON1T3: *“Yes. But still, the doctor is the doctor. The doctor is the doctor, and they expect you to know.”*

There were many examples of ONs trying to get GPs on track, to take responsibility for their part of palliative patient care. They usually found that the GPs complied.

ONS1: *“Actually, when I get a patient referred, I go to the doctor and say now you’re responsible for a sick patient with cancer at home (…) Obviously, sometimes their feathers get a bit ruffled, because I’m so direct. Still, you have to say things the way they are (…). We need updated medicine lists, we need this or that in place and we need a home visit so that when a week later I say she’s getting worse, then you know her baseline.”*

Participants confirmed that there were variations in both GPs’ and nurses’ interest and competence in palliative care, which could lead to patients not receiving optimal care.

ON2T5: *“What I’ve seen at the nursing home is how differently patients are treated, depending on who’s on duty, which nurses and which doctor have seen them and ... as for palliative care, some had had morphine prescribed and were well palliated, while others got next to nothing.”*

## Barriers to effective cooperation between GPs and ONs

There were organizational and cultural barriers to cooperation between GPs and ONs. The two groups often had a different work arrangement. While ONs were salaried and had fixed working hours, GPs were often self-employed, paid fee-for-service and most had busy days seeing patients.

GPT2: *“Let’s put it this way: You could also call or send a message, because sometimes it’s… I’m up to my neck, just too many patients in the waiting room. (…) we have emergencies here, well, we have that all day. (…) So you might start off with four patients and end up with twenty or thirty before lunch, you just don’t know. That makes it very hectic, and unpredictable, compared to a surgery in a big town.”*

ONs often had more predictable days, allowing them to do home visits and attend meetings during normal hours. GPs often chose to do home visits on their way home from work and attend meetings after hours. However, ONs often preferred joint home visits, as in this excerpt from an interdisciplinary FGD.

ON2T3: *“I think it reassures us. As an ON, you have such a great responsibility regarding home death. Actually, a huge responsibility. So when you know that you’re going...that you’ve got a doctor with you, it’s so much easier.”*

GP1T3: *“Then just tell us that you’d like a joint home visit.”*

ON2T3: *“Yes, you can relax much more than when you’re on your own”.*

GP1T3: *“Okay, we’ll let you control this a bit and please tell us (…) Then we have to give it priority, just like an emergency call.”*

ONs clearly identified themselves as belonging to the nursing group, with its common standards and learning strategies. Enhancing their competencies, for example in palliative care, was talked about as a collective project. The ONs all belonged to quality networks, with regular meetings.

ONS2: *“This is also kind of about “making each other better” (...). Because we talk a lot about competence building, but this is also about using our existing competences and improving each other, like “Yes, you can do this” and lifting each other up.”*

GPs, on the other hand, had more individual professional identities and idiosyncratic learning strategies, and rarely referred to collective projects for enhancing their competencies or committing themselves to shared quality standards. Experience from caring for patients was the most common way to learn palliative care. Attending courses was necessary to obtain or renew their speciality, but participation was an individual decision, and meant loss of income.

GPI1: *“I haven’t been on a course in this field, but lots of GPs do take special courses. We’ve had seminars where the university hospital has been here and taught us, which was excellent. So, well, you get experience when you have these patients. (…) In a way, you learn while you’re working.”*

## Discussion

The ideal cooperation between GPs and ONs was a “meeting of experts” with complementary competencies, who discussed and supported each other in their work with palliative patients. When cooperation was deficient, the ONs did not receive the advice and support that they needed from the GPs, while GPs were bypassed by ONs contacting specialists directly. We found significant barriers to cooperation. The first one concerned how primary care is organized and paid for, leading to non-matching time-schedules for the two professions. Secondly, we found that ONs and GPs had different strategies for learning. While ONs belonged to a networking nursing collective, GPs learned mostly from their individual experience of caring for patients.

We had broad, mainly purposive recruitment of 52 health professionals from rural parts of Northern Norway. We obtained rich data from ten FGDs and six interviews. The individual interviews gave in-depth insights into how each professional perceived his or her contribution, while the FGDs were suitable for comparisons and negotiations of relationships. The study gave a mixed picture of cooperation between GPs and ONs, emphasising both positive and negative experiences. The interprofessional FGDs were not planned as interventions; however, in some groups solutions to the problems described by the participants were worked out in real time. Most participants did not have regular interprofessional meetings about palliative care in their local area. Coming together in an FGD seemed to promote frank and constructive interprofessional dialogues, enhancing the distinct and complementary voices of each profession.

We are researchers from two disciplines: BE is an oncology nurse and the head of the Regional Advisory Unit for the Palliative Care Centre, while MLJ is an academic GP. These positions gave us valuable insights, access and credibility, but also influenced our moderator roles, the topics participants chose to talk about and the way the discussions were interpreted. Although the study concerned palliative care and was undertaken in the rural North, we believe that our findings may be transferable to other primary care settings where general practitioners and specialised nurses work together. This study mainly dealt with cancer; however, patients with other diagnoses are also in need of palliative care. To address this, a broader specialisation in palliative nursing has been developed in Norway.

Traditional professional hierarchies did not seem to be a barrier to cooperation in our study. GPs were highly respectful of ONs’ competencies, and welcomed being taught by them [20, 27]. In general, nurses can earn respect and trust from doctors by their clinical experience [28] and competence [27]. The GPs in our study valued the ONs’ contribution to primary palliative care, acknowledging how ONs lightened their working days. With an increasing workload and more complex and severely ill patients in primary care, ONs’ competencies are highly relevant. A concept analysis [29] concluded that “teamwork” concerns a process where healthcare professionals with complementary competencies and common goals exercise concerted effort in patient care, through interdependent collaboration, open communication and shared decision-making. While nurses working in general practice often work on delegation from GPs and do not routinely participate in shared decision-making or goal setting [30], the best examples of collaboration in our study deserve to be called teamwork.

ONs were clear about the medical responsibility being with the doctor. ONs have specialised competence in palliative care, often long clinical experience and many have developed a form of pattern recognition akin to diagnosing. Unlike nurse practitioners, ONs in Norway are not trained in clinical decision-making, which is particularly complex when the patient has comorbidities. They need doctors to discuss with, as these contribute their knowledge of the patient, often from a long-term doctor-patient relationship. Such continuity of care has been identified as a core element of good end-of-life care [31, 32]. In our study, doing home visits together was a way of joining competencies, adjusting clinical judgements and supporting each other. Home visits by the GP are associated with higher satisfaction with end-of-life care [33], fewer visits to the emergency department in the last weeks of life [34] and higher likelihood of patients dying at their preferred place [35, 36].

A recent report depicts GPs as “sidelined” in primary palliative care by nurses and specialists [37]. However, according to the report, all stakeholders want GPs to come back and be key players. As Field already commented on the UK context 20 years ago, “the involvement of specialist providers of palliative care can have detrimental consequences for the generalist expertise of GPs (…) by de-skilling them” [38], and later studies [11, 39, 40] have found similar concerns. In our study, there were examples of ONs bypassing GPs by contacting specialist palliative teams directly. In general, however, this rural study did not confirm the notion of GPs as missing collaborators. Due to distances, rural communities have to be more self-sufficient in health care than urban areas and this might prevent de-skilling of rural GPs in fields like palliative care.

In most areas of Norway, GPs and ONs belong to different organizational structures within primary care, and their working conditions might preclude collaboration. In New Zealand, professionals working in primary care perceived funding models based on fee-for-service as discouraging collaboration, while teamwork was seen to be promoted when practices were funded per capita, and when both nurses and doctors were salaried [41]. Hierarchical business models where GPs own practices and nurses work for them do not favour teamwork [30], whereas promoting the autonomy of each professional can enhance collaboration [42].

## Conclusions

The complementary competencies and autonomous roles of a specialised nurse and a general practitioner represented a good match for primary palliative care. GPs could be valid co-workers for ONs by making maximum use of their generalist competencies. By prioritizing palliative care as much as medical emergencies, as suggested by some participants, and by applying their previous knowledge of the patient, GPs could give ONs much of the crucial support they need. If GPs also gave priority to courses in palliative care [43], that would add value to their expertise. A business model where primary health care is mainly funded per capita could lead to more sustainable practices with several professions sharing patient care. Attention to different professional cultures of working and learning [44] has implications for quality improvement efforts. It is worth noting that some GPs’ surgeries act as communities of practice [45, 46] with common goals and standards. This form of collective organization for learning and quality improvement could be a way forward for the GP profession, and a way to overcome undesirable variations [4, 47] in competence and practice.

## Additional file

Additional file 1: Interview guides. (DOCX 12 kb)

## Abbreviations

DN: District nurse
GP: General practitioner
ON: Oncology nurse with an advanced qualification in palliative care
