# Supplementary material for: Teamwork in primary palliative care: general practitioners’ and specialised oncology nurses’ complementary competencies
Source: BMC Health Serv Res. 2018 Mar 7;18:159. doi: 10.1186/s12913-018-2955-7 (PMC5842567; doi:10.1186/s12913-018-2955-7)
Supplement: Supplementary file 1 — Interview guides. (DOCX 12 kb) [file 12913_2018_2955_MOESM1_ESM.docx]

**Interview guides**

Questions for individual interviews

1. Open start question: Tell me about a patient that you have given palliative care.

2. Follow up questions:

- Re the interprofessional cooperation in palliative care: What works well, and less well?

- How are your professional competencies asked for by other professionals?

- Departing from your competencies - what is your contribution to palliative care?

- To which extent do you feel that your competencies in palliative care are being used?

- Do you feel a need to learn more – and what?

- To which extent are you able to debrief your feelings regarding your work in this field?

Topic guide for focus group discussions

Ask for a patient story as an example of local cooperation

1. Experiences of the existing organisation of palliative care and the cooperation with specialists

2. Experiences of own role in palliative care and in interprofessional cooperation

3. Facilitators and barriers to local cooperation and cooperation with specialist care

4. Discuss need for formalized palliative teams in the local communities

Follow-up questions re:

- local history of palliative care, pioneers

- local geography and culture

- facilitating home death and bereavement care

- palliative care to other patient groups than cancer

- palliative competence, learning, tools in use
